# 

**Published:** 1810-06

**Authors:** 


### To CORRESPONDENTS.

Verax, we trust, will see the propriety of our not inserting *both* his papers, and thereby disappointing some one of our other Correspondents; indeed, that we might be enabled to avail ourselves of all the numerous favours we receive, we sometimes wish that Authors would, as much as possible, avoid redundancy of language, without impairing the value of their observations. It would be the highest satisfaction to us to be distinguished in the Medical World as affording to our Readers, the *multum utile in parvo.*

Censor's letter has been received, and though we cannot insert the *whole,* we will gladly avail ourselves of it with such curtailments as we judge necessary. If Censor objects to our doing so, he will be so good as to inform us by the 10th of the month.

END OF VOL. XXIII.

### INDEX.

ABERNETHY's, Mr. furgical obfervations 242
Abfcefs of the brain, cafe of 376
Acid, effects of muriatic 51
——, on fluoric 292
Adams, Dr. on fmall-pox inoculation 27
—— —— on the hooping cough 177
—— —— on the digeftion of the ftomach after death 399
Addington, Mr. on apparent death 338
Adipofe tumour in a fcirrhous ovary 237
Æther, its ufe in hydrothorax 334
Ague, a remarkable burning one in England 283
Affufion, cold, epilepfy remedied by 20
—— —— fcarlatina anginofa relieved by 413
Air, method of preferving it pure 84
—— pure, beft preventive for fevers 117
Alder, Dr. on the proximate caufes of difeafes 116
—— —— proofs of his theory 281, 469
Alimentary canal, cafe of difordered 431
Alkalies, their action on animal concretions 47
Alps, on the ftructure of the 525
Amenorrhœa, obfervations on 238
America, difeafes in 287
Amputation, obfervations on 108
—— —— remarks on 329
Andrews's, Dr. account of vaccination at Madeira 85, 93
Aneurifms, obfervations on 242
Angina maligna, a fatal difeafe among cattle 285
Angina pectoris, prize queftion on 260
Animal œconomy, effects of heat on the 307
Animals, on the accidents arifing from the bite of rabid 41
——, on the torpidity of 502
Ankers, Mr. on purulent ophthalmia 155
Antimony, tefts to difcover the glafs of 439
Apartments, method for heating 152
—— —— plan for erecting fpacious 262
Apothecaries, cautions to 67
—— ——, on the practice of 139
Arminger, Mr. on difeafes of the army 159
Army, on the fever of the 155
—— medical board, change in the 438
Arfenic, on the effects of 238
Arfenic, cafe of poifon by 384
——, on detecting the prefence of 448
Arteries, on tying the 252
Artery, on tying the 479
Afhford's, Dr. tabular views of the human body 514
Atmofphere, temperature of the 256
Auftrian government, prize queftions of the 352
Bailey's, Mr. cafe of abfcefs of the brain 376
——, Mr. cafe of uterine hemorrhage 493
Barbadoes, remarkable difeafe in 168
Barton, Dr on the torpidity of animals 502
Bath, advantages of the cold 118
Bed, defcription of a portable 338
Beddoes, Dr. on digitalis 241
Belladonna, effects of the 455
Bile, probable ufes of the 248
Bilious difeafes, on 512
Birds, on the migration of 502
Bifmuth, virtues of the white oxidule of 501
Bladder, obfervations on the 453
Blifters, their ufe in enteritis 171
Blount's, Mr. communication from French journals 497
Body, effects of the folar heat on the human 193
——, tabular views of the human 514
Bones, folution in the parietal 273
——, on the exfoliation of the 456
BOOKS, CRITICAL ANALYSIS OF NEW MEDICAL.——A Treatife on Champignons, by M. Paulet, M. D. 68. Facts eftablifhing the efficacy of the opiate Friction, by M. Ward, 71. The Phyfician's Vade Mecum, by R. Hooper, M. D. 75. Remarks on the Purulent Ophthalmy, by J. Ware, 77. Identities afcertained; or an Illuftration of Mr. Ware's Opinion, 79. An Effay on Winter Cough and Confumption, by T. Buxton, M. D. 150. The Edinburgh Journal, Nos. XXI & XXII, 154, 237, 428, 516. Surgical Obfervations, by John Abernethy, 242. Enquiry into the Nature, Caufes, and Cure of Hydrothorax, by L. Maclean, M. D. 330. Annual Report of the Humane Society for 1809, 336. Experiments on the Foot of the living Horfe, by

Bracy Clarke, 338. A Scientific and popular View of the Fever of Walcheren, by J. B. Davis, M. D 418. A new Family Herbal, by R. Thornton, M. D. 424. Treatife on Cheltenham Waters, &c. by T. Jamefon, M. D. 508 Tabular Views of the Human Body, by H. Afhford, M. D. 514
BOOKS, NEW MEDICAL, ANNOUNCED. Dr. Adams's Edition of Hunter on the Venereal Difeafe 86. Dr Trotter's Appeal to the Officers of the Royal Navy, *ib.* Dr. Uwins's Remarks on the Caufes of Fever, 87. Mr. Clarke on the ftructure of the Horfe's Foot, 175 Dr. Maclean on Hydrothorax, 176. Mr. Afhford's Epitome of Anatomy, *ib.* Mr. Travers on Injuries in the Canal of the Inteftines, *ib.* Mr. Stocker's Tranflation of the London Pharmacopœia, *ib.* Meffrs. Kirby and Spence's Introduction to Entomology, 262. Dr. Latham's Facts and Opinions concerning Diabetes, 263. Mr. Lee's Effay on Mortification, *ib.* Dr. Smith's Inftructions for Gouty and Rheumatic Perfons, *ib.* Dr. Trotter's Effay on Drunkennefs, 264. Dr. Watfon on the Inftruction of the Deaf and Dumb, *ib.* Dr. Bradley's firft Lines of the Practice of Phyfic, 352. Mr. Ramfden on Derangements of the Tefticle, 440. Dr Rees' Practical Obfervations on Spafms of the Stomach, 528. Dr Stock's Life of Dr. Beddoes, *ib.* Dr. Toulmin's Elements of the Practice of Medicine, *ib.*
Borland's, Dr report of army difeafes 183
Boffen, account of the finking of the ifle of 263, 526
Bowel complaints, prevalence of 349
Bowels, cafe of conftipation of the 501
Brain, an inflammatory action of the 83
——, cafe of fuppuration of the 89
——, defcription of the plate to 192
——, cafe of abcefs of the 376
Burnett's, Mr. report on the difeafes of the army 164
Burns, Mr. on the digeftion of the ftomach 428
———— anfwer to 399
Buxton, Dr. on cough and confumption 150
Cancer, cafe of 500
Candles, new manufacture of tallow for 261
Cantharides, on the ufe of 311
Cameron's, Dr. cafe of fecondary fmall-pox 122
Carbonate of potafh, ufe of 45
Carrots in ulcers, efficacy of 140
Cat, a child bitten by a 460
Cataplafms, impropriety of warm 392
Catarrh, treatment of 172, 259, 435
Cattle, remarkable difeafe in 166
Chamberlain's, Mr. cautions 67
Champignons, account of a treatife on 68
Chaptal's, M. experiments on colours 84
Cheltenham waters, obfervations on the 508
Chevalier's, Mr. cafe of fuppofed rabies 223
Chincough, on the 348, 436
Chifholm, Dr. on lues bovina 166
Clapton, meteorological diary kept at 437
Clark, Mr. on the horfe's foot 175
Clarke's, Dr. medical report at Nottingham 154
———— cafe of 165
Cobweb, medicinal virtues of 161, 212
Cold affufion in epilepfy, efficacy of 20
———— in fcarlatina anginofa, effects of 413
Coley's, Mr. cafe of fcirrhous ovary 237
Colours, experiments on 84
Concretions, action of alkalies on animal 47
Confcripts, regulations in France refpecting the 429
Confumption, obfervations on 150
Contagion, prize queftion on 260
Contagious difeafe, account of a 33
Convulfion, obfervations on 369, 480
Cornea, cafe of ulcerated 57
Correfpondents, to 88, 176, 352, 440, 528
Corunna, on the treatment of the fick returned from 517
Cotton, effects of fwallowing a fkein of 431
Cough, on the winter 150
——— on the contagious property of hooping 178
Coup de Soleil, cafes of 196
Cramps, virtues of oxidule of bifmuth in 501
Crawford's, Dr. cafe of hepatic affection 226
Croup, cafes of 172
Cynanche maligna, a remarkable 166
———— tonfillaris, obfervations on 348
Darwin, Dr. on the theory of 332
Davies, Mr. on obstetrical cafes 13
———— obfervations in reply to 290
Davis, Dr. on the fever of Walcheren 418
Death, on the digeftion of the ftomach after 399, 428
Delaroche, M. on the effects of heat 307
Depletion, extraordinary cafe of 175
Defcroizelles, M. on the pickle of violets 351
Deffaignes, M. on fpontaneous phofphorefcence 387
Diarrhœa, obfervations on 434
Dickfon, Dr on the ufe of ftimulants, &c. 392

Diemerbroeck, on an opinion of 28
Digeftion of the ftomach after death, on 399, 428
Digeftive organs, on diforders of the 249
Digitalis, its efficacy in hydrothorax 276
Difeafe, account of a contagious 33
Difeafes and cafualties in London 87
——— in an eaftern diftrict of London, reports of 173, 349, 436
——— at Edinburgh, reports of, 81, 170 257, 347, 433, 521
——— on the proximate caufes of, 116, 469
——— of the heart, on 273
Diftention, on lymphatic 300
Dogs, cafes of horfes bitten by 1
———, experiments on 43
Dropfy, effects of the digitalis on 279
——— cured by the vapour bath 501
Druggifts, on the practices of 24, 139
Dupin's, M. account of a contagious difeafe 33
Earle's, Mr. cafe of fuppuration of the brain 89, 192
Earthquakes, confequences of 281
Economy, effects of heat on the animal 307
Edinburgh, report of difeafes at 81, 170, 257, 347, 43[illegible], 521
——— Journal, critical analyfis of 154, 237, 428, 516
———, difference in the college of 212
Edmonfton, Dr. on elephantiafis 516
Edwards, Mr. on inflammation of the inteftines 54
——— on ulcerated cornea 57
——— on hydropthalmia 125
Elaterium, on the ufes of 335
Elephantiafis, obfervations on 516
Emetics hurtful in remittent fevers 498
Enteritis, treatment of 171, 258
Entomology, account of a new work on 262
Epidemics, hiftory of 281
Epidemic difeafe, account of an 455
Epilepfy, cafe of, with obfervations 20
——— treated with opiate frictions 74
Errata 264, 440
Eruptive difeafe, defcription of an 441
Efcarotics, treatment of ganglia by 432
Experiments on rabid animals 41
——— on carbonate of potafh 45
——— on champignons 70
——— for preferving a pure air 84
——— on colours *ib.*
——— on expectorated matter 214
——— on fluoric acid 292
——— chemical 350
——— on the potatoe fly 325
——— on the effects of heat 307
——— on the foot of the horfe 339
——— on the pickle of violets 351
Experiments on the effects of arfenic 384
——— on fpontaneous phofphorefcence 387
——— on the glafs of antimony 439
Eyebright, great virtues of 104
Febrile difeafes, efficacy of opiate friction in 71
Fever, on the general caufes of 121
———, remarks on the infantile 128
——— of the army from Spain, obfervations on the 155
——— of Walcheren, fcientific view of the 418
——— at Edinburgh, prevalence of 435
Fevers, on emetics in remittent 498
Fincham's, Mr. cafe of difordered alimentary canal 431
Fire-places, improvement in 152
Fifhes, on the refpiration of 451
Fluoric acid, on the decompofition of the 292
Fluor albus, remedy for 501
Fly, on the properties of the potatoe 323
Fœtus, account of a remarkable 84
Fogo, Dr. on hydrocephalus 25
———, Mr. on the uterus 518
Fontaine, M. la, on plica polonica 204
Fofbrooke, Mr. on the difeafes of the army 159
Fofter's, Mr. meteorological diary 437
Foxglove, obfervations on the 241
France, medical report of the inftitute of 451
——— hydrophobia prevalent in 525
Fretis, Dr. on vaccination at Madeira 99
Friction, on opiate 71
Galvanifm, experiments in 292
Gamboge, its ufe in hydrothorax 335
Ganglia, on the treatment of 432
Gerard, Dr. on tetanus rabienfis 41
Gimbernat, treatment of crural hernia on the plan of 379
Glafs of lead and antimony, tefts to difcover 439
Gonorrhœa, obfervations on 78
Goodlad, Mr. on purulent ophthalmia 154
Gorget, apology for the cutting 238
Gorham, Dr. on the potatoe fly 323
Gourlay, Dr. on vaccination at Madeira 98
Gout, remarkable effects of the loadftone in the 174
——— medicine, defcription of a 527
Gregory, Dr. his difpute with the college 212
Grenada, remarkable difeafe at 166
Gum of opium, mode of preparing the 351
Guy's hofpital, circular letter of the phyfical fociety at 174
——— lectures at 175
Hamilton, Dr. on ipecacuanha 318

Hargrove's, Mr. cafe of folution in the parietal bones 273
Harrifon's, Dr. bill, remarks on 63, 137
Hawes, Dr. character of 337
Haygarth, Dr. reply to 469
Headach, ufe of ftannum in 315
Heart, cafe of difeafe of the 242
——— cafe of organic difeafe of the 265
Heat, on the effects of folar 193
——— its effects on the animal economy 307
Hecla, eruptions of mount 283
Hemorrhage, on fuppreffing 253
——— on uterine 290, 461
———, cafe of uterine 493
Hepatic affection, cafe of 226
Herbal, account of a new family 424
Hernia, cafe of omental 246
——— fpecific for the cure of 261
——— cafe of ftrangulated crural 379
——— method of reducing crural 456
Heywood's, Dr. cafes of difeafed uterus 299
Hill, Mr. on the effects of arfenic 238
Hooper's, Dr. Phyfician's Vade Mecum, account of 75
Hooping-cough, on the contagious property of the 178
Horfes bitten by a dog, cafes of 1
———'s foot, obfervations on the 175, 338
Howard, Mr. on glafs of lead 439
Howe's, Mr. obfervations on amputation 108, 479
——— defcription of fea fcurvy 233
Howfhip's, Mr. obfervations on tetanus 15
——— on folar heat 193
——— obfervations on convulfions 369, 480
Humane fociety, report of the 336
Humboldt, M. on refpiration 451
Hume, Mr. on arfenic 443
Hydrocephalus, on theories of 25
——— cafes of 171
——— remarks on cafes of 286
Hydropthalmia, cafe of 125
Hydrophobia, cafes of 1
———, effay on 41
———, obfervations on 73
———, analogy between tetanus and 149
———, cafes of 154
———, account of a cafe of fuppofed 223
———, Mr. Kidd on 459
———, prevalent in France 525
Hydrothorax, on the nature and cure of 330
———, cafes of 276
Hypnum Crypfum, obfervations on 525
Infantile fever, remarks on 123
Inflammation of the lungs, on 151, 349
Inflammatory difeafes, on 170, 434
Inoculation, on fmall-pox 27
Intelligence, medical, 84, 174, 259, 350, 438, 525
Inteftines, cafe of inflammation of the 54
Ipecacuanha, on the pernicious effects of 199, 318, 447
———, obfervations on 447
Iritis, obfervations on 501
Ifchia, on the warm baths of 498
Ifland, difappearance of an 263, 526
Jackfon, Dr. on the virtues of eyebright 104
Jamefon, Dr. on the Cheltenham waters 508
Jones, Dr. on tying the arteries 253
———, Mr. on poifon by arfenic 384
Kali preparatum, its ufe in hydrothorax 334
Kidd, Mr. on hydrophobia 459
Kirby's, Mr. work on entomology 262
Knowles's, Mr. method of preferving leeches 321
——— cafe of plica polonica 368
Lead, tefts to difcover glafs of 439
LECTURES, MEDICAL ANNOUNCED.— Dr. Buxton, on Medicine, 86. Dr. and Mr. Clarke, on Midwifery, *ib.* Dr. Clutterbuck, on the Theory and Practice of Phyfic, *ib.* 440, 528. Dr. Ramfbotham, on Midwifery, *ib.* Dr. Reid, on the Theory and Practice of Medicine, *ib.* 528. Dr. Squire, on Midwifery, *ib.* Mr. Taunton, on Anatomy and Surgery, *ib.* At St. Thomas and Guy's Hofpitals, 175. Dr. Hooper, on the Theory and Practice of Phyfic, 176. Dr. Adams, on Pathology and Therapeutics, 263, 440. Dr. Clough, on Midwifery, *ib.* Dr. Roberton on ditto, 440. Mr. Carpue on Anatomy and Surgery, 528.
Leech, remarkable cafe of a 498
Leeches, obfervations on 212
———, method of preferving the 321
Leg, obfervations on ulcers of the 432
Lempriere, Dr. on the malady at Walcheren 183
Leprofy, account of an epidemic 284
Loadftone, its virtues in gout 174
London, difeafes and cafualties in 87
———, account of difeafes in an eaftern diftrict of 173, 349, 436
———, anniverfary meeting of the medical fociety of 352
Lues bovina, obfervations on 166, 430
Lungs, obfervations on inflammation of the 151, 349
Luffac, M. on the fluoric acid 292
Lyall, Mr. on purulent ophthalmia 154
Lymphatic diftention of the lower extremities, on the 300
Maclean's, Dr. enquiry into hydrothorax 330

Maclean's, Dr. cafes of ditto 276
Madeira, ftate of vaccination in 85, 93
M'Grigor, Dr. on the fever of the army 155
Magnefia, good effects of carbonate of 477
Magnet, its virtues in gout 174
Malady at Walcheren, report on the 183
Matter, obfervations on expectorated 214
Mansfield, chief juftice, his opinion 190
Meafles, on the treatment of 348
Medical reform, on 48, 63, 209
——— profeffion, bill for improving the 188
——— fociety of Paris, proceedings of the 259
——— fociety of London, meeting of the 352
——— practitioners, on the rank of 137
——— board, on the new army 438
Meneges, Dr. on vaccination 102
Mercury, on the ufe of 520
Meteor, account of a remarkable 286
Meteorological diary 437
Morveau, M. on carbonate of potafh 45
Muriatic acid, effects of 51
National inftitute, medical report of the 451
——— vaccine eftablifhment, report of the 114
Nervous affections, ufe of ftannum in 315
Nofology, obfervations on 127
Nottingham, medical report for 154
Obftetrical cafes, obfervations on 13
Ophthalmy, remarks on the purulent 77, 79, 154
Opiate friction, efficacy of 71
Opium, its ufe in fevers 176
——— method of preparing the gum of 351
Organic difeafe of the heart, cafe of 265
Organs, on the digeftive 247
Ofgood's, Dr. uterine cafe 462
Ovary, cafe of a fcirrhous 237
Parietal bones, folution in the 273
Paris, Dr. on muriatic acid 51
———, meeting of the medical fociety at 259
Paroletti, M. on colouring matters 350
Paulet's, M. treatife on champignons 68
Pearfon, Dr. on expectorated matter 214
Peck, Mr. on the leech worm 212
Penis, account of an eruptive difeafe on the 441
Pepys, Sir Lucas, his letter to the fecretary at war 438
Phofphorefcence, on fpontaneous 387
Phthifis pulmonalis, connection between amenorrhœa and 238
Phyfiology, obfervations on 451
Placenta prefentation, on 14
Plague, hiftorical notices of the 282
Planetary influence, on the 476
Plica polonica, obfervations on 204
——— ——— prize queftion on 260
——— ——— cafe of 288
Pneumonia, cafes of 171
Poifon, account of a vegetable 454
Pole's, Dr. meteorological ftatements 255
Potafh, on the ufe of carbonate of 45
Potatoe fly, on the veficating properties of the 323
Practitioners, on the rank of medical 137
Prefcriptions, on the preparation of medical 24
Publications, new medical 88, 176, 264
Purges, on the abufe of 500
Quackery, on the ftate of 64
Rabid animals, obfervations on 41
Rabies, fuppofed cafe of 223
Rattle-fnake, on the bite of a 85
Re-agents, on chemical 350
Reform, medical, obfervations on 48, 63, 134, 137, 209, 378
——— ——— intended bill for 188
Refpiration, obfervations on 451
Roberton's, Dr. reports of difeafes 81, 170, 257, 347, 433, 521
——— remarks on the cafes of 286
——— ufe of cantharides 311
Royfton, Mr. on an eruptive difeafe 441
——— on ipecacuanha 447
Saffron, defcription of the meadow 426
Saunders, Mr. account of the death of 264
Scarlatina anginofa, cafes of 52
———, obfervations on 413
Scirrhous ovary, cafe of 237
Scrotum, a remarkable tumour in the 61
Scurvy, defcription of the fea 233
Seamen, on the food of 237
Shearman, Dr. on amenorrhœa 238
——— on ftannum 314
Sheep, origin of the rot in 283
Shoulder joint, on amputation at the 329, 479
Simmons, Mr. on the cutting gorget 238
Small-pox, on inoculation for the 27
———, cafe of fecondary 123
———, prevalent at Edinburgh 172, 258
Snails a cure for hernia 261
Solar heat, its effects on the body 193
Solution of the parietal bones 273
Sores, efficacy of carrots in 140
Spain, difeafes of the army from 155
Spallanzani, on the difcoveries of 451
Spafmodic difeafes, on opiate frictions in 71
Spence's, Mr. intended work on entomology 263
Spencer, Mr. on hydrocephalus 286
Spider's web, virtues of 161, 212
Stag, account of a tame 85
Stannum, virtues of 314
Stimulants, on the ufe of 393

Stomach, its digeftion after death 399
Stoves, on heating rooms by 152
Sullivan, governor, cafe of 265
Superfœtation, on the doctrine of 278
————, obfervations on 457
Suppuration of the brain, cafe of 89, 192
Surgeons, ftate of the college of 263
Surgical examination 440
——— obfervations 242
Surr, Mr. on horfes bitten by a dog 1
Swallows, on the migration of 504
Sympathy, on organic 245
Symphifis pubis, fection of the 499
Tallow, improved manufacture of 261
Tape-worm, a remedy for the 84
Tarenne's, M. fpecific for hernia 261
Taunton's, Mr. cafe of difeafed tefticle 59
Temperature, on a well regulated 150
————, average of the 255
Tefticle, account of a difeafed 59
Tetanus, obfervations on 15
——— rabienfis, effay on the 41
——— analogy between hydrophobia and 149
Tetanus, cafe of 518
Thenard, M. on fluoric acid 292
Thermometrical ftatements 255
Thomas's Hofpital, lectures at 175
Thoracic vifcera, economy of the 22
Thornton's, Dr. family herbal, account of 424
Torrence, Mr. on fcarlatina anginofa 413
Towfey, Mr. on ipecacuanha 417
Tumour in the fcrotum, a remarkable 61
Turnips a cure for ulcers 148
Turpentine a remedy for the tape-worm 84
Twins, a cafe of 457
———— obfervations on *ib.*
Typhus, cafe of 53
Udiometrical ftatements 255
Ulcerated cornea, cafe of 57
Ulcers, efficacy of carrots in 140
——— on the treatment of 392
——— of the leg, obfervations on 432
Upas tree, on the poifon of the 454
Urethra, on ftrictures in the 78
————, cafe of imperforate 122
Urinary organs, on difeafes in the 45
Uterine hæmorrhage, obfervations on 290, 461
——— cafe 462
——— hæmorrhage, cafe of 493
Uterus, cafes of difeafed 299
——— on the functions of the 518
Vaccination at Madeira, account of 85, 93
———— remarks on 111
———— circular letter on 114
Van Marum, Dr. on purifying rooms 84
Vauquelin's, M. improved tallow 261
Vetch, Dr. on ophthalmia 78
Vincent, Mr. on preparing gum of opium 351
Vinegar in hydrophobia, on 460
Violets, ufe of the pickle of 351
Viper, remedy for the bite of the 456
Vifcera, economy of the thoracic 22
Walcheren, account of the malady at 183
Walker, Dr. on thoracic vifcera 22
———— on the virtues of carrots 140
Ward, Mr. on opiate friction 71
———— on hydrophobia and tetanus 149
Ware, Mr. on purulent ophthalmy 77
———— remarks on 79
Warren's, Dr. cafe of organic difeafe of the heart 265
———— cafe of crural hernia 379
Weather, on the influence of the 475
Webb, Mr. on ulcers of the leg 432
Webfter's, Mr. hiftory of epidemics 281
———— defence of 469
Willey, Dr. on hooping cough 179
Wine in fevers, obfervations on 116
Winter cough, on the treatment of 150
Wifhart's, Mr. cafe of difeafe of the heart 242
Woodham, Mr. on the treatment of ganglia 432
Woolriche, Mr. on vaccination 103
Worms, remedy for 84
Wyer, Dr. on lymphatic diftention 300

### REFERENCES TO THE PLATES.

Mr. EARLE's Case of Suppuration of the Brain – – – 89

Mr. ROYSTON's History of an acute Eruptive Disease of the Integuments of the Penis – – – – – – – 441

